# Transcriptomic Profiling of Central Nervous System Regions in Three Species of Honey Bee during Dance Communication Behavior

**DOI:** 10.1371/journal.pone.0006408

**Published:** 2009-07-29

**Authors:** Moushumi Sen Sarma, Sandra L. Rodriguez-Zas, Feng Hong, Sheng Zhong, Gene E. Robinson

**Affiliations:** 1 Neuroscience Program, Institute for Genomic Biology, University of Illinois at Urbana-Champaign, Urbana, Illinois, United States of America; 2 Department of Entomology, University of Illinois at Urbana-Champaign, Urbana, Illinois, United States of America; 3 Department of Animal Sciences, University of Illinois at Urbana-Champaign, Urbana, Illinois, United States of America; 4 Department of Statistics, University of Illinois at Urbana-Champaign, Champaign, Illinois, United States of America; 5 Department of Bioengineering, University of Illinois at Urbana-Champaign, Urbana, Illinois, United States of America; University of California Davis, United States of America

## Abstract

**Background:**

We conducted a large-scale transcriptomic profiling of selected regions of the central nervous system (CNS) across three species of honey bees, in foragers that were performing dance behavior to communicate to their nestmates the location, direction and profitability of an attractive floral resource. We used microarrays to measure gene expression in bees from *Apis mellifera, dorsata* and *florea*, species that share major traits unique to the genus and also show striking differences in biology and dance communication. The goals of this study were to determine the extent of regional specialization in gene expression and to explore the molecular basis of dance communication.

**Principal Findings:**

This “snapshot” of the honey bee CNS during dance behavior provides strong evidence for both species-consistent and species-specific differences in gene expression. Gene expression profiles in the mushroom bodies consistently showed the biggest differences relative to the other CNS regions. There were strong similarities in gene expression between the central brain and the second thoracic ganglion across all three species; many of the genes were related to metabolism and energy production. We also obtained gene expression differences between CNS regions that varied by species: *A. mellifera* differed the most, while *dorsata* and *florea* tended to be more similar.

**Significance:**

Species differences in gene expression perhaps mirror known differences in nesting habit, ecology and dance behavior between *mellifera, florea* and *dorsata*. Species-specific differences in gene expression in selected CNS regions that relate to synaptic activity and motor control provide particularly attractive candidate genes to explain the differences in dance behavior exhibited by these three honey bee species. Similarities between central brain and thoracic ganglion provide a unique perspective on the potential coupling of these two motor-related regions during dance behavior and perhaps provide a snapshot of the energy intensive process of dance output generation. Mushroom body results reflect known roles for this region in the regulation of learning, memory and rhythmic behavior.

## Introduction

Animal brains are composed of anatomically distinct regions which are further made up of spatially and functionally coherent populations of neurons and glia. They specialize in processing different kinds of signal input from the animal's internal and external environment and integrate the information to mount an appropriate physiological and behavioral response. Even though many molecular processes are considered universal to all cells, transcriptomics and *in situ* hybridization analysis have revealed extensive localized regulation of genes expressed in the brain in both vertebrates and invertebrates [1]–[3]. Studies of mammals and song birds have revealed strong connections between brain-region specific gene expression and behavior [4], [5].

The brain of the honey bee, *Apis mellifera*, is among the best studied insect brains, from neuroanatomical, neurochemical and neurophysiological perspectives [6]–[8]. In addition, numerous brain-region specific analyses of gene expression exist for the honey bee, but they are largely limited to analyses of single genes via *in situ* analysis [9]–[11]. Although honey bees have been used for several large-scale analyses of behaviorally related gene expression at the whole brain level [12]–[14], large-scale transcriptomic comparisons of different brain regions in the bee brain have not yet been conducted. This information would be helpful to our understanding of how known regional differences in structure and function in the bee brain relate to behavioral regulation.

We performed the current study with two goals in mind. Firstly, to carry out a transcriptomic profiling of selected regions of the honey bee brain to determine the extent of regional specialization in gene expression. A recent neuroanatomical analysis [15] of dance language [16], the famous communication system used by honey bee foragers to communicate to their nestmates the location, direction and profitability of an attractive food source they encounter in the environment, suggested that multiple brain regions are involved in the perception and production of dance communication, meaning that regional analysis of brain gene expression will be required to understand this remarkable system. Therefore, our second goal was to explore the honey bee CNS at the transcription level to get a picture of how the different regions might contribute to the behavioral output associated with dance communication.

Honey bee foragers need to carry out a spectrum of sensory information processing not only to navigate but also to produce the dance language. These include visual information about the landscape and location, direction information, measurement of distance, measurement of gravity to name a few. Based on previous neuroanatomical and behavioral studies in honey bees and other insects, we know that the following CNS regions are likely to be involved in sensory processing and regulation of dance: 1) the optic lobes (OL), which receive sensory input from the compound eyes and the ocelli and are comprised of 3 distinct neuropils, the lamina, medulla and lobula [17]–[19]; 2) the mushroom bodies (MB), which consist of intrinsic neurons called Kenyon cells [20], [21] and a complex neuropil arranged into anatomically defined subparts strongly associated with olfactory learning, higher order visual processing, multi-modal sensory integration and general arousal [22]–[29]; and 3) the central brain (CB), which contains (among other neuropils) the central complex [30], a precisely arranged array of neurons implicated in the control of acoustic communication and coordinated movements during courtship in *Drosophila* (fruit fly) and gomphocerine grasshoppers [31]–[33], orientation to polarized light [34], [35]. We also included the second thoracic ganglion (TG) because it innervates and controls the body parts involved in the dance output namely, the wings, the middle and hind legs, muscles of meso and metathorax and the articulation of the abdomen with the thorax through the propodeum [36]. The TG has also been implicated in coordinating motor patterns, generating rhythmic movements in flies and crickets and gregarious behavior in locusts [37]–[39].

We exploited the striking differences in dance language that exist in the genus *Apis* [16], focusing on three species, *A. mellifera, A. dorsata*, and *A. florea*. *A. mellifera*, the cavity nesting Western honey bee, the model honey bee species for which we have the genome sequence and related genomic resources [40], is the species in which the dance language was first described. The other two species that are confined mostly to South Asia show some striking differences in the dance language [16]. Our previous study showed differences in gene expression between these species [13], but the study was conducted on whole brains, and more importantly, it compared foragers and one-day-old bees, so it was not clear to what extent the differences were related to differences in dance behavior or differences in behavioral maturation.

We generated CNS region-specific profiles of gene expression for *A. mellifera, dorsata*, and *florea* individuals sampled directly from beehives while they were engaged in dance behavior. We were particularly interested in testing for two types of patterns of CNS regional gene expression in association with dance behavior. Differences in gene expression between brain regions that are *consistent* across the three bee species should reflect intrinsic functional specialization within the *Apis* nervous system. By contrast, regional differences that are *different* across the three bee species (region by species interactions) may reflect differences that are related to species differences in behavior.

## Methods

### Sample collection and processing

**Figure 1:**
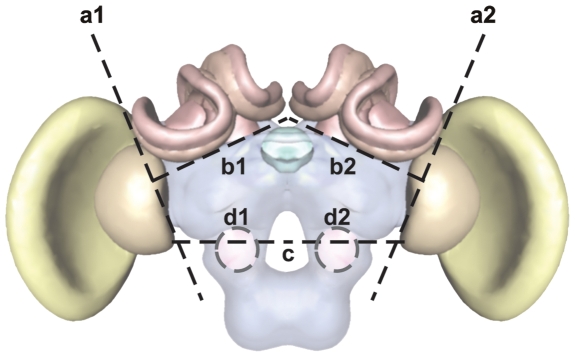
Schematic representation of brain with the regions that were used in the study. Dotted lines show the meridians of separation between the regions: a1 and a2–optic lobes, b1 and b2–mushroom bodies, c , d1 and d2 mark the lines along which the sub-esophageal ganglion and the antennal lobes were removed. Brain schema in Figure 1, 2, 3 and 5 drawn after [71].

Dancing bees returning from successful pollen collecting trips were easily identified on honeycombs according to established criteria [41] and collected from 2–4 natural colonies on location in Bangalore, India between 9 AM and 12 PM each collection day. Individuals were collected on liquid nitrogen and subsequently stored in ultra-low freezers. Samples were shipped on dry ice to the University of Illinois and stored at −80°C until processed further. 2 colonies from each species were used for subsequent analysis. Frozen brains were fixed in RNALater ICE (Ambion/Applied Biosystems, Austin, Texas) and dissections were carried out on fresh ice under a stereomicroscope (Olympus SXZ12). Fig. 1 shows the meridians along which the brain was divided to give the 3 brain regions studied. Due to limitations of the technique the divisions were not precise and might have missed cell bodies that lie at the junction of two regions, e.g. some cell bodies that lie close to the antennal lobes and send their projections into the central complex might have been removed along with the antennal lobes [30]. However, a majority of the cells that belong to a particular region were included. In order to include the central complex in the central brain region, we could only have the calyces of the mushroom bodies in the MB region. However, the calyces contain the cell bodies of the intrinsic Kenyon cells [20] where most (but not all) transcription takes place.

Extractions were carried out with RNAeasy (Qiagen, Valencia, California) kit and quantified using a Nanodrop™ spectrophotometer (Thermo Scientific, Wilmington, Delaware). 100 ng of each RNA sample was amplified using the MessageAmp kit (Ambion/Applied Biosystems, Austin, Texas). Amplified mRNA from OL, MB, CB and TG of each individual dancer (11–12 individuals/colony/species) were used in labeling and hybridization as in previous studies [13].

### Microarray analysis

We analyzed 4 CNS regions of 72 pollen dancers of 3 species on an *A. mellifera* brain EST microarray. This array has been shown to perform well for these species even though it was designed with *mellifera* sequences [13]. A loop design was employed for microarray analysis [42], with each CNS region compared to another region belonging to the same species, on multiple arrays per species. A total of 117 arrays were used in this study, each probing equal quantities of amplified mRNA (2 ug). CNS regions from individual bees were hybridized on each array.

### Data Analysis

Microarray data generated in this study meet Minimum Information about Microarray Experiment (MIAME) standards and are available at ArrayExpress [43] under accession number E-TABM-700. A total of 117 arrays were used for statistical analysis, after quality control analysis. Microarray features that received a “−100” flag by the scanning software GenePix or that had a median fluorescence intensity <300 were removed from the analysis [44]. Gene expression measurements were log2-transformed and normalized using a LOWESS smoothing function. Microarray elements with missing information in more than two arrays or control sequences [44] were removed from the analysis. Data from duplicated spots were averaged and adjusted for global dye and microarray effects [45], [46]. In order to minimize errors and the occurrence of false positives, only genes that were expressed at detectable levels in at least 115 arrays of the quality tested 117 microarrays were included in the data analysis. Thus 5182 or 74% of the genes on the arrays that passed the filter criteria can be considered to be ubiquitously expressed throughout the honey bee CNS, irrespective of species. The dataset for each species was then analyzed in two ways, separately subject to ANOVA (ANOVA 1) and combined in a single dataset before being subject to an ANOVA (ANOVA 2).

A linear mixed effect ANOVA model was used to describe the normalized expression intensity (y_jklmn_ or y_ijklmn_) on a gene-basis: ANOVA 1: y_jklmn_ = μ+R_j_+D_k_+A_l_+B_m_+H_n_+ε_iklmn_; ANOVA 2: y_ijklmn_ = μ+S_i_+R_j_+SR_ij_+D_k_+A_l_+B_m_+H_n_+ε_ijklmn_ where μ denotes the overall mean, S_i_ denotes the effect of the ith species, R_j_ denotes the effect of the jth region, D_k_ denotes the kth dye, A_l_ denotes the effect of the lth array, B_m_ denotes the effect of the mth array batch, H_n_ denotes the effect of the nth bee, and ε_jklmn_ or ε_ijklmn_ denotes the residual. The terms H_n_, A_l_ and e_jklmn_ or e_ijklmn_ were treated as random effects and the remaining terms were treated as fixed effects. Statistical tests were based on a global variance model (F3). The false discovery rate criterion was used to adjust for multiple testing [47]. Statistical analyses were conducted using the SAS statistical package.

Results of a subsequent post-hoc t test were then used to carry out the subsequent pattern analysis. Using a cut-off p value of 10^−4^ we coded a negative expression ratio (log2 fold change) between any two regions as −1, while a positive expression ratio was coded as 1. A non-significant expression difference was coded as 0. Expression profiles that compared all six possible contrasts MB-CB, CB-OL, CB-TG, MB-OL, MB-TG and OL-TG were then used to cluster genes using a K-means clustering program. Contrasts that compared MB with another region gave the best clustering outcome and therefore only those 3 contrasts CB-MB, OL-MB and TG- MB were used for subsequent pattern analysis. 27 possible patterns of gene expression profiles are possible in these 3 contrasts as summarized in Table 1. Depending on the expression profiles that the genes had in each species, they were grouped into one of the 27 patterns. GO enrichment analysis of genes showing a pattern of interest was carried out with a Chi-square test with Yates continuity correction [12]. Since this correction results in a conservative estimate of the p value, we used a cut-off of p = 0.01 for statistical significance. At this threshold, the number of false positives expected was several times lower than the actual significant results obtained. For example, out of 4590 comparisons that were carried out for genes that were upregulated in any one CNS region compared to another (irrespective of species), 262 GO terms were identified at the significance level of p = 0.01, which is more than 5 times of the expected number of false positives (45.90) .

**Table 1:** The number of genes that showed each of 27 possible expression patterns.

Pattern#	Pattern			Count of genes in species			In both species			In all 3 species
	CB_MB	OL_MB	TG_MB	AM	AD	AF	AM = AD	AM = AF	AD = AF	AM = AD = AF
1	−1	−1	−1	450	513	525	319	333	349	276
2	−1	−1	0	42	56	25	17	8	7	5
3	−1	−1	1	1	0	0	0	0	0	0
4	−1	0	−1	337	334	165	148	87	73	53
5	−1	0	0	52	63	76	10	4	4	2
6	−1	0	1	0	1	0	0	0	0	0
7	−1	1	−1	1	4	0	1	0	0	0
8	−1	1	0	0	3	0	0	0	0	0
9	−1	1	1	0	0	0	0	0	0	0
10	0	−1	−1	57	75	62	21	9	16	7
11	0	−1	0	136	171	97	51	28	28	16
12	0	−1	1	13	5	0	0	0	0	0
13	0	0	−1	253	192	115	60	22	16	4
14	0	0	0	2177	1889	2894	1392	1767	1566	1205
15	0	0	1	327	292	188	93	51	44	22
16	0	1	−1	11	22	0	2	0	0	0
17	0	1	0	228	330	165	109	70	87	51
18	0	1	1	94	102	85	22	22	18	9
19	1	−1	−1	0	0	0	0	0	0	0
20	1	−1	0	2	5	0	0	0	0	0
21	1	−1	1	8	15	0	3	0	0	0
22	1	0	−1	1	1	1	0	1	0	0
23	1	0	0	80	80	82	11	10	9	2
24	1	0	1	482	482	244	233	118	96	64
25	1	1	−1	1	1	0	1	0	0	0
26	1	1	0	62	62	42	18	9	12	7
27	1	1	1	367	367	416	181	208	206	132

−1 denotes gene expression is higher in MB compared to the other region being compared, 0 denotes equal expression levels, while 1 denotes lower expression level in MB compared to the other region being compared. Abbreviations: CB = central brain, MB = mushroom bodies, OL = optic lobe, TG = thoracic ganglion; AM = *A. mellifera*, AD = *A. dorsata*, AF = *A. florea*.

## Results

### CNS-specific differences in honey bee gene expression consistent across the species

A total of 5182 genes representing 74% of the genes present on the array passed through our analysis filters (see Methods). About half the genes showed no CNS-specific pattern of expression, presumably reflecting genes involved in processes common to all nervous tissue, across all three species. There were significant differences in gene expression between CNS regions for ca 50% of the genes (ANOVA 1, FDR<0.001; 2597 in *mellifera*, 2777 in *dorsata* and 2028 in *florea*, Table S1). Approximately 50% of these have been annotated, largely on the basis of known functions in *Drosophila melanogaster* [40]. The MB was most different from the other CNS regions in gene expression and was thus a major contributor to this region effect. The average proportion of genes differentially expressed in MB was 72% compared to CB (1837 in *mellifera*, 1949 in *dorsata* and 1580 in *florea*), 60% compared to OL (1482 in *mellifera*, 1663 in *dorsata* and 1333 in *florea*) and 82% compared to TG (2177 in *mellifera*, 2204 in *dorsata* and 1704 in *florea* = 1704;). By contrast, the smallest difference in gene expression was observed between CB and TG. The average proportion of genes differentially expressed in TG compared to CB was 14% (461 in *mellifera*, 422 in *dorsata*, and 225 in *florea*).

Similar results were obtained in an independent clustering-based analysis that generated 27 distinct patterns of expression differences between the different CNS regions (Table 1). The biggest gene cluster group (pattern #14) was comprised of genes that showed no region-specific pattern of expression. These genes again presumably reflect genes involved in processes common to all nervous tissue, across all three species. As in the analysis above, ca. 50% of genes showed this pattern in each species (1205 genes). More genes in this category were shared between *mellifera* and *florea* than either did with *dorsata*. Patterns 1 and 27 were the next major groups, wherein MB had a higher or lower expression level respectively compared to the other regions. Again 50% of genes with these patterns were shared between the three species. Genes expressed at similar levels in MB compared to OL but differentially expressed compared to CB and TG were part of the next two major patterns (nos. 4 and 24).

**Figure 2:**
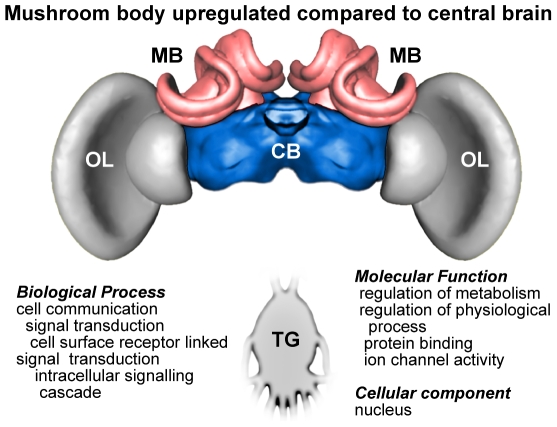
Results of GO enrichment analysis of genes that showed consistent differences in gene expression across the three honey bee species in the mushroom bodies compared to central brain.

**Figure 3:**
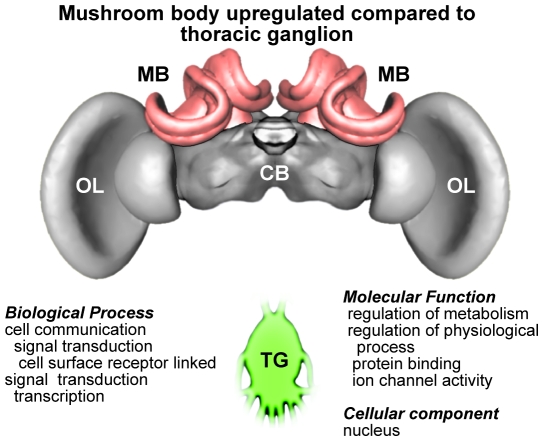
Results of GO enrichment analysis of genes that showed consistent differences in gene expression across the three honey bee species in the mushroom bodies compared to thoracic ganglion.

To gain further insight into the possible functional significance of the consistent differences in gene expression between CNS regions across the three species, we performed GO enrichment analyses on the groups of (GO annotated) genes that showed a directional bias of expression in one region compared to another in all 3 species. As with the previous analyses, results for MB compared to CB and TG yielded the most coherent patterns, while comparisons with OL or comparisons between OL, CB and TG did not show concordance between species. Figs. 2 and 3 summarize the results of the enrichment analysis of genes that were upregulated in MB compared to CB and TG, respectively. An almost identical list of GO terms appeared in both comparisons, reflecting consistent themes for MB across the three species. Many of the enriched GO categories pertain to neuronal activity while other categories include those involved in cell surface receptor-linked signal transduction and intracellular signaling cascades, and genes that bind to other proteins (GO molecular function: protein binding).

The following are among the genes upregulated in MB compared to CB and TG in all three species that are known (primarily from functional analysis in *Drosophila*) to be involved in synaptic transmission: *Inositol tris-phosphate receptor* (*Itp-r83a*), known to be preferentially expressed in mushroom bodies of honey bees by *in situ* hybridization analysis [48], *Ryanodine receptor* (*Drosophila* ortholog *Rya 44F*), *Nicotinic acetylcholine receptor* and *Muscarinic acetylcholine receptor*; and *Cacophony*, a calcium channel gene whose protein product is involved in synaptic transmission that is implicated in *Drosophila* courtship behavior and adult locomotion, particularly adult male courtship song [49]. The following are among the genes upregulated in MB compared to CB and TG in all three species that are known to be involved in signal transduction: *Shaggy, CAMKII* known to be highly expressed in honey bee mushroom bodies by *in situ* hybridization analysis [50], *Pka-R2* and *Pka-c* code for the regulatory and catalytic subunits of cAMP dependent protein kinase or PKA. *Shaggy* codes for a crucial protein kinase in *Drosophila* and is an important developmental gene that is also involved in regulation of circadian rhythms in the adult [51]. PKA plays an important role in development and is also involved in adult learning and memory [52], [53]. It is expressed at higher levels in the honey bee mushroom bodies compared to the rest of the brain [54]. *Calcium/calmodulin-dependent protein kinase II* or *CAMKII* is involved in learning and memory, specifically long term memory and courtship behavior [55].

In contrast to these results for the MB, we did not detect any concordance in enriched GO categories for genes that are upregulated in OL compared to CB or TG across species. Furthermore, comparatively fewer genes (51 out of 502) in these comparisons showed similar patterns across the species.

### CNS-specific differences in honey bee gene expression that vary by species

**Figure 4:**
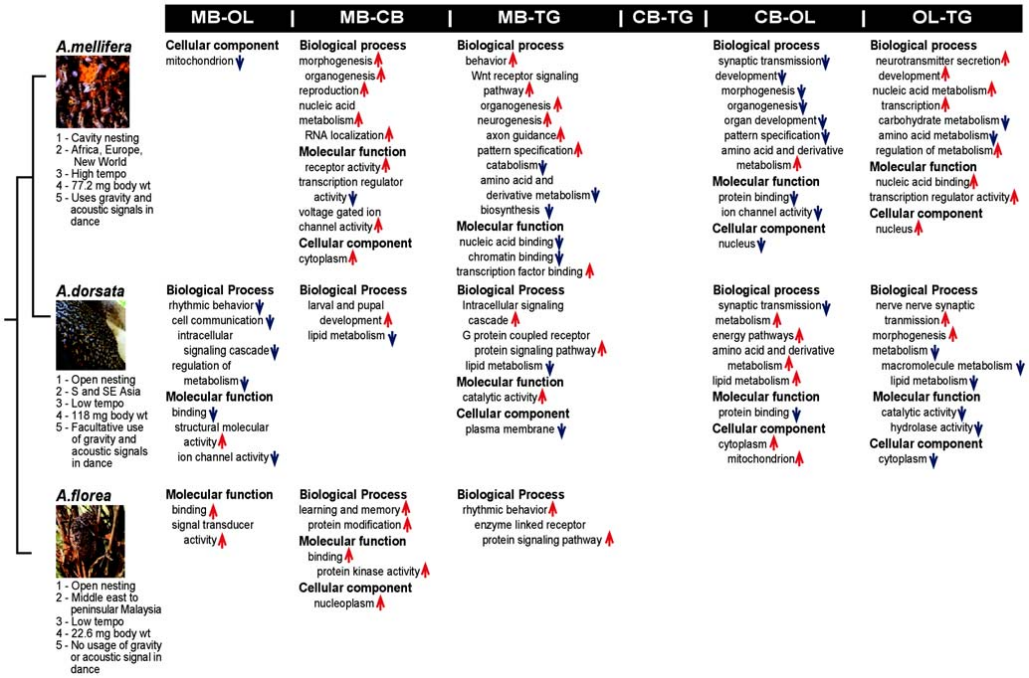
Results of GO enrichment analysis of genes that showed species by CNS region differences in gene expression, based on pair-wise comparisons of the CNS regions (p<0.01, Chi-Square test with Yates continuity correction). First column shows the species with relevant differences in behavior and ecology with phylogenetic ranking after [68], [69]. Upward arrows indicate upregulation of enriched genes of a given GO class in the first brain region of the pair, while downward arrows indicate upregulation of enriched genes of a given GO class in the second brain region of the pair. Abbreviations as in Table 1.

There were significant CNS region by species interactions in gene expression for ca 14% (709 of 5182) of the genes (ANOVA 2, FDR<0.001; Table 2, Table S1). These reflect regional differences in gene expression that are *different* across the three bee species. These genes were then subject to a GO enrichment analysis (see Methods). Fig. 4 summarizes cases where there were differences between species in the GO classes that were enriched in genes upregulated in one CNS region compared to another (ANOVA 1). Consistent with the lack of across-species concordance for MB-OL comparisons, there were numerous cases of species-specific MB-OL differences. For example, genes upregulated in OL compared to MB in *dorsata* were greatly enriched for a number of GO classes that denote involvement in intracellular and cell-cell signaling and regulation of metabolism. On the other hand, *mellifera* only showed an enrichment of mitochondrial genes upregulated in OL compared to MB while *florea* by contrast, showed an enrichment of signal transduction genes upregulated in MB compared to OL.

**Table 2:** Genes showing species by CNS region interaction at p<0.001.

Species\Region	CB	MB	OL	TG
**AD_AF**	524	516	506	520
**AD_AM**	541	534	515	523
**AF_AM**	556	554	542	575

Genes were compared using a post-hoc t-test for differences in expression profiles (p<0.05) for a given CNS region between 2 species. Numbers that showed significant differences are summarized below. Abbreviations as in Table 1.

**Figure 5:**
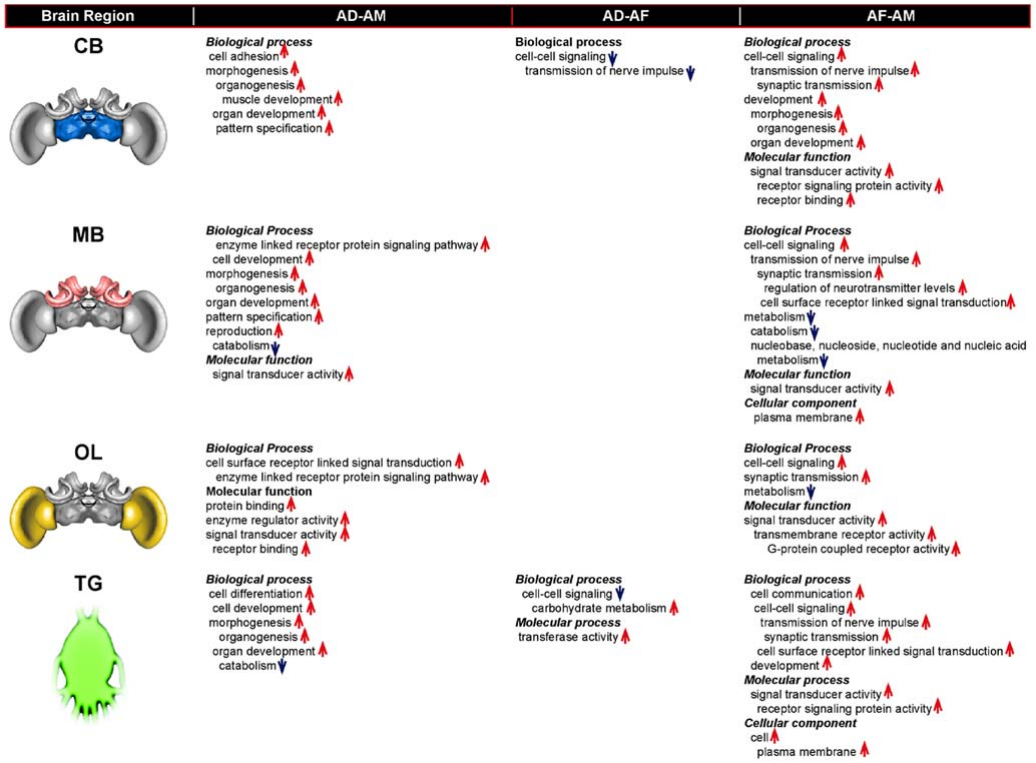
GO enrichment analysis of genes that showed significant differences in expression between species for a given CNS region highlighted in the brain schematic (p<0.04, Chi-Square test with Yates continuity correction). Upward arrows indicate an overrepresentation of upregulated genes of a given GO class in the first species of the pair, while downward arrows indicate overrepresentation of upregulated genes of a given GO class in the second species of the pair. Abbreviations as in Table 1.

Fig. 5 summarizes the bias in GO enrichment of genes that were differentially expressed in a given region of one species compared to another species (ANOVA 2). The most biased enrichment was observed primarily for comparisons of *mellifera* CNS regions with corresponding regions in *florea* and *dorsata*. There were many more GO categories for enriched genes upregulated in *florea* and *dorsata* CNS regions compared to corresponding regions in *mellifera*, (32 out of 36 and 26 out of 28 categories enriched in genes differentially expressed in *florea* and *dorsata* respectively, compared to *mellifera*). This is in contrast to the 5 GO categories enriched in *florea* and *dorsata* comparisons.

## Discussion

This “snapshot” of the honey bee CNS during dance behavior revealed some insights into how behavioral differences between species might be reflected in gene expression. The first insight that we gained was that the mushroom bodies were very different from the other CNS regions studied and consistently showed the biggest differences in terms of gene expression. In all three species, the mushroom bodies were the most different from the other regions in terms of gene expression. In addition, genes involved in signaling and synaptic remodeling were seen to be upregulated compared to other CNS regions. Results from GO analyses highlight the function of the mushroom bodies in learning and memory, with enrichment in categories such as transcriptional regulation and ion channel activity, among others. These results are consistent with known roles for the mushroom bodies in the regulation of rhythmic behavior, learning and memory [28], [29], [33], [56], [57]. In addition, our results nicely correspond with earlier *in situ* hybridization data and immuno-staining data for genes like *Inositol tris-phosphate receptor*, *CAMKII* and PKA that were also shown to be highly expressed in mushroom bodies [48], [50], [54].

Our molecular data provide suggestive evidence for the mushroom bodies being an integration or “association” area in the honey bee CNS [58]. Since we have sampled bees while they were dancing, we are perhaps looking at that part of the CNS that plays the biggest role in processing sensory information and coordinating the dance output. It has been already shown that the small-type Kenyon cells of the mushroom bodies show prominent neural activity in foraging and dancing honey bees [57]. However, 2 alternate possible explanations must also be considered. Firstly, the mushroom bodies are the largest pair of neuropils in the honey bee brain containing 35% of neurons in the honey bee brain. They integrate information from various sensory modalities and thus play a central role in the insect brain [6]. Although we have controlled for the discrepancy in cell numbers between the different regions (see Methods), it is possible that the transcription pattern obtained in mushroom bodies reflects the multimodality of neurons and sensory processing in this part of the CNS. In other words, we are perhaps looking at a chronic difference between mushroom bodies and other parts of the CNS in the honey bee that has nothing to do with the behavior that was being executed at the time of sampling. A third possibility is that the expression profile of honey bee mushroom bodies might be diagnostic of insects in general that have structurally complex mushroom bodies like the hymenopterans (ants, bees and wasps), dictyopterans (cockroaches) and coleopterans (scarab beetles) [59]. Although not closely related, these insects share a marked flexibility in food acquisition behaviors.

Unfortunately, studies on other insects are insufficient for adopting or rejecting any of the 3 scenarios detailed above. There are only two other transcriptomic profiling studies of insect CNS regions and both were carried out on insects that have simpler and smaller mushroom bodies compared to honey bees, *Drosophila melanogaster* (fruit fly) [60] and *Schistocerca gregaria* (locust) [61]. Additionally, the animals in those studies were reared in the laboratory and not sampled while carrying out a specific behavior unlike our focal animals. Our approach, applied to other species, might be very useful in exploring the functional significance of region-specific expression in the brain and relating it to evolutionary constraints.

There were far fewer instances of common gene regulation in the optic lobes across the three species. Evidence in other insects links body size to visual ability [62], [63], so the visual systems of the three honey bee species we studied could also be different due to marked differences in size [64]. Of the three species, only *dorsata* has the ability to fly in very low light conditions. Perhaps reflecting this special ability, the optic lobes showed enrichment of upregulated genes involved in intracellular and cell-cell signaling and regulation of metabolism.

We did not compare dancers with bees carrying out other behaviors because a previous study in honey bees showed that behaviors that are not temporally or physiologically well separated are also not well separated by gene expression [65]. As foragers are very different from workers that stay in the nest [12] the most logical comparison would have been foragers that dance with foragers that do not dance. However this distinction is often ephemeral and not chronic and perhaps more appropriate for quantitative proteomics [66]. Nevertheless, our study provides some hints into the neural and molecular workings of dance behavior. The similarities in gene expression between the central brain and thoracic ganglion provide a unique perspective on the coupling of these two regions during dance behavior. The central brain receives multisensory input like the mushroom bodies does and also coordinates locomotion and rhythmic movement, while the thoracic ganglion receives motor signals from the central brain and provides motor output to the wings, legs and abdomen while generating complex movement patterns [37], [38], [67].

GO analysis reveals that, genes upregulated in both the central brain and thoracic ganglion were similar, mostly dealing with metabolism and energy production. It is likely that these findings reflect the energy intensive process of motor signal transmission and neuronal firing that would be required in generating dance output. If this speculation is correct, then at least some parts of our “snapshot” reflect brain activity that is actually related to dance behavior, rather than to behavior that is regulated over a longer time scale, such as other aspects of foraging behavior. If so, it is worth noting that the two species that showed the most differences in gene expression in the central brain and thoracic ganglion, *mellifera* and *florea*, are also the two species that show the biggest differences in dance “dialects,” i.e., the precise relationship between dance movements and the distance to the food resource that they encode. This speculation suggests that the central brain and thoracic ganglion gene lists may be particularly valuable for providing candidate genes for distance-related aspects of dance communication.

*Apis florea* and *dorsata* showed more CNS-region-specific similarities in gene expression when compared to each other, and both showed more differences when compared to *mellifera*. This cannot be attributed to evolutionary distance since recent phylogenetic analyses suggest that all three species are separated by 8–10 million years [68], [69]. Instead, we speculate that this reflects the similarities in nesting habit, ecology and dance behavior that exist between *florea* and *dorsata*, and not *mellifera* (Fig. 4) [64]. Both *florea* and *dorsata* are open nesting bees that build a single honeycomb from a support, in contrast to *mellifera*, which is cavity nesting and builds multiple parallel honeycombs inside a tree cavity. Both *florea* and *dorsata* are endemic to South Asia and found in primarily tropical and subtropical ecosystems while *mellifera* is a Western honey bee that is found in both temperate and tropical environments. The dance language also shows striking differences, with *florea* dancers communicating reportedly exclusively in the visual modality while *dorsata* is able to use both visual and acoustic signals in its dance communication facultatively. *mellifera* on the other hand, constrained by the darkness of its hive, communicates with acoustic and vibrational signals. Genes that were upregulated in *florea* and *dorsata* central brain compared to *mellifera* were enriched in GO classes morphogenesis, organogenesis and organ development while genes whose products have signal transducer activity were enriched in *florea* and *dorsata* mushroom bodies compared to *mellifera* mushroom bodies. Probing these classes of genes in future studies might lead to a deeper understanding of the molecular basis of species differences in *Apis*.

In another promising result, genes that were upregulated in *mellifera* mushroom bodies compared to *florea* or *dorsata* were primarily involved in metabolism while genes enriched for catabolism were downregulated in *florea* and *dorsata* mushroom bodies compared to *mellifera* mushroom bodies. This result closely mirrors our earlier transcriptomic analysis of forager and one-day-old bees that also showed differences among these species in brain expression of metabolism genes [13]. Dyer [70] reported that *mellifera* colonies show higher rates of colony activity or “worker tempo” than *florea* or *dorsata* and have a higher colony metabolic rate. We speculate that to the extent that brain metabolism reflects whole organism metabolic activity our molecular results might in some way reflect these behavioral differences. Four genes involved in metabolism that showed species differences in both studies are alpha mannosidase (*α-Man(II)b*), *Lethal (3) neo18*, a serine-type carboxypeptidase (CG4678) and *Ebony*.

In addition to the four genes mentioned above, 34 other genes showed species differences in expression in our earlier study and species by CNS region differences in the present study. Some of the more obviously behaviorally related genes include orthologs of the *Drosophila* genes *Doubletime* (*Dbt*, also known as *Discs overgrown*), *Synaptotagmin* (*Syt*), *Synaptotagmin IV* (*SytIV*) and *slowpoke*. These genes are involved in circadian rhythms (*Dbt, slowpoke*) which figure prominently in dance behavior; [15] and synaptic activity and motor control (*slowpoke* and Synaptotagmins). They also provide good candidate genes to explore the molecular basis of dance language.

## Supporting Information

Table S1 ESTs and Gene IDs of honey bee genes from Anova 1 and 2 that had significant differences at FDR<0.001
(0.55 MB XLS)
